# Key points of technical review for the registration of SARS-CoV-2 antigen/antibody tests

**DOI:** 10.4155/bio-2020-0219

**Published:** 2021-01-11

**Authors:** Hongran Li, Jingyun He, Wen Bao, Peirong Wang, Yunfeng Lv, Chao Xu, Peng Hu, Yu Gao, Shengwei Zheng, Juanjuan An, Gang Deng, Jinchun Dong

**Affiliations:** ^1^Center for Medical Device Evaluation, NMPA, Building 1, No. 50 Qixiang Road, Haidian District, Beijing 100081, PR China

**Keywords:** antibody, antigen, registration, SARS-CoV-2, tests

## Abstract

Coronavirus disease-2019 (COVID-19), caused by the novel severe acute respiratory syndrome coronavirus 2 (SARS-CoV-2), has spread globally since its first report and become a worldwide pandemic. In response to the outbreak of COVID-19, Center for Medical Device Evaluation, NMPA (CMDE) initiated emergency review and approval procedures to accelerate the process of reviewing emergent medical products and issued the Key Points of Technical Review for the Registration of SARS-CoV-2 Antigen/Antibody Tests (Key Points) to provide the requirements on the technical review of the tests. With uncontrolled spread and evolution of COVID-19 in the world, continuous prevention and measurements are necessary for fighting this pandemic and SARS-CoV-2 antigen/antibody tests are still urgently needed. This article is an attempt to expand clarification of the Key Points to wider audiences based on current understanding of SARS-CoV-2 to facilitate the development and application of SARS-CoV-2 antigen/antibody tests.

A novel coronavirus pneumonia (coronavirus disease-2019, COVID-19), caused by a novel severe acute respiratory syndrome coronavirus 2 (SARS-CoV-2, originally named 2019-nCoV), has spread globally and rapidly since its first report [1–4]. The WHO declared this outbreak as a Public Health Emergency of International Concern on 30 January 2020 [5] and characterized COVID-19 as a pandemic on 11 March 2020 [6]. As of 1 November 2020, there have been 45,942,902 confirmed cases of COVID-19, including 1,192,644 deaths globally according to the report from WHO [7]. This pandemic has still not been controlled effectively. Most of the patients with COVID-19 have mild or moderate disease, however up to 5–10% present with severe and even life-threatening disease course. Although different treatments are being evaluated, the most important way to control this pandemic is the development of an effective and safe vaccine widely available [8].

SARS-CoV-2 taxonomically belongs to the Sarbecovirus subgenus of the coronavirus family and is the seventh member of human coronavirus [9]. Four of these human coronaviruses, HCoV-229E, HCoV-NL63, HCoV-OC43 and HCoV-HKU1, usually cause mild-to-moderate symptoms and the other two human coronaviruses, severe acute respiratory syndrome coronavirus (SARS-CoV) and the Middle East respiratory syndrome coronavirus (MERS-CoV), generally cause more severe cases [10]. The SARS-CoV-2 genome is around 30 kb and contains *ORF1ab* (encoding polyproteins PP1ab and PP1a), structural proteins genes, which encode surface glycoprotein (S), an envelope protein (E), membrane protein (M), nucleocapsid (N) proteins and several accessory proteins [11].

Rapid, accurate and sensitive detection for SARS-CoV-2 is essential for the prevention of the COVID-19 pandemic and the treatment of COVID-19 patients. Real-time reverse transcription PCR (RT-PCR) has been used as gold standard for SARS-CoV-2 RNA detection. But in past clinical practice and research, the false negative results of nucleic acid tests may occur due to different factors, such as patient sampling quality, viral load and distribution, and the standardization of the detection process among others [12]. Reliable serological tests can provide more information about SARS-CoV-2 infection, and SARS-CoV-2-specific IgG and IgM have been used as one of the evidences for COVID-19 diagnostics [13]. But what should be noted that the positive rate of SARS-CoV-2-specific IgM and IgG is low in the first week since symptoms onset, and usually the serological tests can not directly diagnose the presence of the virus [13]. It just can be used as a supplementary method to increase the positive rate of the diagnosis for suspected cases with negative SARS-CoV-2 nucleic acid results and can also help to confirm the disease status [12].

In order to respond to this pandemic effectively, the National Medical Products Administration of China (NMPA) initiated the emergency review and approval procedures soon after its outbreak to accelerate the approval of SARS-CoV-2 tests. The Center for Medical Device Evaluation (CMDE) drafted the ‘Key Points of Technical Review for the Registration of SARS-CoV-2 Antigen/Antibody Tests’ (hereinafter referred to as the Key Points) on 11 February 2020 and issued it on 25 February [14] to provide a guidance for reviewer and commercial manufacturers. This Key Points is consistent with the Provisions for *In-Vitro* Diagnostic Reagent Registration of China (2014) [15] and provides requirements and methods for evaluation of SARS-CoV-2 antigen/antibody detection reagents, which mainly includes analytic performance (such as limit of detection, inclusive test, cross reactivity validation, etc.) and clinical trial requirements. Based upon this Key Points, 25 SARS-CoV-2 antibody and two SARS-CoV-2 antigen tests have been approved by NMPA (as of 10 November 2020), all of which are applied by Chinese manufacturers and play important roles in fighting against COVID-19 pandemic in China.

Since the regulatory requirements for SARS-CoV-2 products are different among different regulatory agents, correct and comprehensive understanding of each guidance will improve the design, development and application for marketing or emergency use during this pandemic. With the uncontrolled spread and evolution of SARS-CoV-2 in the world, continuous prevention and countermeasures are necessary, and comprehensive explanation of the requirement for SARS-CoV-2 tests are needed for wider audiences. In addition, continuous updated understanding of SARS-CoV-2 and COVID-19 put forward the necessity for analysis of the suitability of this Key Points issued at the beginning of this pandemic for current situation. This article is an attempt to provide clarification of the Key Points to wider audiences based on the current understanding of SARS-CoV-2 to facilitate the development and application of SARS-CoV-2 antigen/antibody tests.

## Scope of application

The Key Points is applicable for SARS-CoV-2 antigen/antibody assays, which are used to conduct *in vitro* qualitative detection of SARS-CoV-2 antigen/antibody in the samples of serum, plasma, whole blood, throat swab, bronchoalveolar lavage fluid, sputum or other respiratory secretions. Semi-quantitative and quantitative assays are not included because there is no international standard reference material to demonstrate them. It is also difficult to be verified by clinical trials according to limited understanding of clinical significance of quantitative and semi-quantitative tests [16]. Currently, only qualitative assays have been approved for SARS-CoV-2 antibody detection. Since the Key Points is to provide the requirement for the application of SARS-CoV-2 antigen/antibody assay, the clinical intended purpose must be clear. With deep understanding of SARS-CoV-2 and its clinical significance, the semi-quantitative and quantitative assays may also be included in the guidance.

## Analytical performance evaluation

According to Provisions for *In-vitro* Diagnostic Reagent Registration of China (2014) [15], the analytical performance of candidate tests should be evaluated and verified, which includes limit of detection, inclusivity, analytical specificity, precision, hook effect and other suitable performance. Since the reagents used for research in the lab are quite different from those produced in a real production environment in many aspects, such as personnel, environment, operation and production batch, and the performance may not be represented by each other, the reagents used for performance evaluation should be manufactured under the control of quality management system. The analysis report submitted to CMDE should include detailed information such as research aim, materials and methods, results, statistical analysis and conclusion. It is suggested to focus on the following items.

### Sample collection & processing

Correct sample collection and processing is very important for correct SARS-CoV-2 assay. Sample collection by individuals and by medical providers may lead to very different results. Although each collection method has its own advantages, here only sample collection by healthcare provider is discussed. The factors of disease course, clinical symptoms and medication treatment should be considered when determining the collection time point of clinical samples. For swab specimens, the requirement of sampling swab and sample preservation solution (sampling solution) should be clarified, including sampling swab material (including swab head and swab rod), sample container and sample solution (such as composition, concentration and dosage of sample solution). For blood samples, anticoagulant should be investigated. The sample collection is suggested to be conducted in accordance with the Technical Guidelines for Laboratory Detection of SARS-CoV-2-infected Pneumonia [17].

### Limit of detection

Limit of detection (LoD) studies determine the lowest detectable concentration of SARS-CoV-2 antigen or antibody. According to the principle of method for LoD evaluation in EP17-A2 [18], the determination of LoD for the SARS-CoV-2 IgM or IgG antibody assays can be performed by detecting a serial of dilutions from samples with known antibody concentration and these dilutions are tested in replicates by the measurement procedure in which results are judged to be negative or positive. Each dilution should be divided into three to five copies and no less than 20 replicates are required for each copy. For each dilution, a ratio is computed as the number of replicates with a ‘positive’ outcome per the total number of replicates tested. The antibody level with 90%–95% positive detection rate should be used as the LoD. Antibody titer should be confirmed by suitable method. For antigen detection, the difference is that the sample containing the virus instead of antibody is diluted for the LoD detection. Three representative clinical samples from different source or virus stock from culture should be used for LoD analysis.

The established LoD should be verified using at least three virus strains or clinical samples with temporal and regional characteristics, which are different from those used for LoD determination. The concentration should be equal to the claimed LoD and the positive detection rate should reach 90–95%. The consistence of diluents with the matrix of applicable sample type will decrease the matrix effects. Negative samples can be used for dilution. For antigen detection, detailed confirmation method for virus titer, the method for virus identification and the results of samples should be provided. For antibody detection reagent, it is required to provide detailed methods and verification results of antibody type and titer.

### Inclusivity

Inclusivity refers to the ability to detect different types of pathogens to be tested, including known types, pathogen subgroups (groups), serotype and genotype.

Five major groups (G_614_, S_84_, V_251_, I_378_ and D_392_) of SARS-CoV-2 viral populations have been defined [19] and the relationship between Gly614 replacement in the spike protein and increased infectivity has been reported [20–23]. This feature of genetic diversity and rapid evolution of SARS-CoV-2 virus might induce conformation change of proteins and probably lead to the antigenicity change [24]. Whether the diversity affects the performance of tests should be evaluated carefully.

The seroconversion of different types of antibody displayed distinct features according to several studies [12,25] and the time of seroconversion may vary between individuals [26]. The first seroconversion day of IgA has been reported at the second day since the onset of the initial symptoms, while for IgM and IgG it was reported at 5 days after onset. After 32 days since symptom onset, the seroconversion rate for IgA, IgM or IgG reached 100% [25]. IgM and IgG specific to N and S proteins have been reported to increase gradually after symptom onset and can be used for detection of SARS-CoV-2 infection [27]. The accuracy of antibody tests for people infected with COVID-19 varied at different time points since the onset of first symptoms, which was 30% at the first week, increased to 70% after 2 weeks and reached the highest in week 3 (more than 90% detected) [28]. These features of different types of antibodies request the detailed analysis for the samples collected at different time and different regions.

In order to evaluate the capacity of proposed assay to detect different isolates of SARS-CoV-2, the real clinical samples of COVID-19 patients from different sources with temporal and regional characteristics should be verified. It is difficult to obtain more clinical samples for so many manufacturers to verify the performance in such an emergent situation. Ten different clinical samples derived from COVID-19-positive patients with positive SARS-CoV-2 antibody collected at different times and from different places are regarded as capable for verifying the inclusivity. Those COVID-19-positive cases with negative real-time RT-PCR results should also be included if they are confirmed by criteria outlined by ‘*Guidelines for the Diagnosis and Treatment of Coronavirus Disease 2019 (8th Trial Version)*’ in China [13]. More samples may add burden on the manufacturer and delay the development process and accessibility of tests during an emergent period. So at least ten different samples are recommended for detection of antigens, IgM antibody and IgG antibody and the LoD and repeatability should be studied. The concentrations of SARS-CoV-2 antigen, IgG or IgM in clinical samples since the symptom onset or PCR positive without symptoms, as well as the determination methods, should also be provided

Although total antibodies (combination of IgA, IgG and IgM) have been used as targets of SARS-CoV-2 antibody assay, the function and clinical meanings of IgA itself still remain unclear. IgA has not been regarded as one of the evidences for COVID-19 diagnostics according to ‘*Guidelines for the Diagnosis and Treatment of Coronavirus Disease 2019 (8th Trial Version)*’ in China [13]. But with more and more investigation of IgA, the indication of IgA test can clarify and related information can be updated.

### Analytical specificity

Analytical specificity reflects the ability of an assay to detect solely the measurant [29], including cross reactivity and interference. The specificity of the assay depends on the specific recognition of the SARS-CoV-2 antibody and antigen. For an immunoassay, special attention should be paid to the possibility of the cross reactivity or the recognition of nontarget components, which is different from inclusivity. For inclusivity, the measurant is a target antigen or antibody for SARS-CoV-2 virus in samples from different regions or different times. If the assay is unable to detect all of the types of SARS-CoV-2, false negative results may occur. But for specificity, non-SARS-CoV-2 pathogens with the potential to give false positive results should be investigated.

#### Cross reactivity

The cross reactivity for related pathogens should be verified. The sequence homology of SARS-CoV-2 and other human coronaviruses in their respective spike proteins make them most likely cross-reactive with each other and strong cross-recognition between SARS-CoV-2 and SARS/MERS-CoV antibody has been reported [30]. Individuals infected with influenza A and B may have similar symptoms as those infected with SARS-CoV-2 and coinfection of SARS-CoV-2 and influenza [31,32] or other common respiratory pathogens has been reported [33]. Immunopathological similarities between COVID-19 and influenza has been indicated [34].

In order to evaluate the specificity of tests comprehensively, the cross reactivity of SARS-CoV-2 antigen, IgM antibody or IgG antibody tests should be verified respectively. According to the multiple respiratory pathogens related guidance [35] and the opinion of experts, the following potential cross reactants are suggested: antigens of or antibodies against endemic human coronavirus (HKU1, OC43, NL63 and 229E), SARS coronavirus and MERS coronavirus; H1N1 (novel influenza A H1N1 virus 2009, seasonal H1N1 influenza virus), H3N2, H5N1, H7N9, influenza B Yamagata, Victoria, respiratory syncytial virus, rhinovirus group A, B and C, adenovirus 1, 2, 3, 4, 5, 7 and 55, enterovirus group A, B, C and D, Epstein–Barr virus, measles virus, human cytomegalovirus, rotavirus, norovirus, mumps virus, varicella zoster virus and Mycoplasma pneumoniae.

The source, negative/positive, species/type and concentration/titer confirmation of antigen, antibody and other samples used for verification of cross reactivity shall be provided. For antigen tests, medical decision concentrations of viruses, such as 10^5^ plaque forming unitor/ml (PFU/ml) or higher, are recommended for cross-reactive verification. For antibody tests, the methods and results for antibody confirmation should be clarified.

The cross reactivity between IgG antibody and IgM antibody against SARS-CoV-2 with high concentration should be verified. No less than 20 normal human samples should also be evaluated.

What should be noted that this list is just a requirement for the premarket application of SARS-CoV-2 antigen/antibody detection tests and does not provide all of the potential cross-reactive pathogens. More and more evidence indicated that SARS-CoV-2 infection can lead to multiple organ failure, especially lungs, heart, kidney, gastrointestinal and hepatic system, brain and skin [36,37] and coinfection of SARS-CoV-2 and other pathogens have been reported [38,39], such as HIV [40] and hepatitis virus [41] etc. So potential cross reaction in such situations should be considered during clinical utility.

#### Interference of endogenous/exogenous substances

The potential interference of related endogenous/exogenous substances should be evaluated for the assay. Substances recommended can be referred to in the issued Key Points [14]. The potential highest concentration of interfering substances (regarded as ‘worst case’ conditions) and the weak positive concentration of virus antigen and antibody are recommended for the interference evaluation. If streptavidin–biotin system is used for the assay, the interference of free biotin in samples [42,43] should be analyzed carefully.

### IgM antibody class specificity

At least five samples containing SARS-CoV-2-specific IgM antibody should be evaluated for class specificity. The detection for the samples treated with certain chemicals (e.g., 2-mercaptoethanol or dithiothreitol) should give a negative result.

### Precision

According to EP05-A3, precision means the closeness of agreement between indications or measured quantity values obtained by replicate measurements on the same or similar objects under specified conditions [44]. It includes repeatability, intermediate precision, and reproducibility, which indicates the measurement precision under a set of repeatability conditions, intermediate precision conditions, or reproducibility conditions of measurements seperately [44].

Applicants should set up acceptable criteria, including the standard deviation or coefficient of variation, for the evaluation of precision. Multiple factors, such as detection time, equipment, operator, location, run and other elements, should be considered for the precision analysis. Reasonable evaluation period should be set that at least 20 or 5 days according to different experiment designs. The detailed information can be found in the EP05-A3 document [44]. Consequently, between-batch/within-batch, between-day/within-day and between-operator precision can be evaluated comprehensively.

The clinical samples of at least three levels of concentration should be analyzed for precision evaluation, which are negative samples, positive samples near the cutoff and (moderate/strong) positive samples. The appropriate precision requirements should be set in accordance with product characteristics.

For negative samples, the concentration of analytes is lower than LoD or equals to zero and the percentage of negative results should be 100% (n ≥ 20). For positive samples near the cutoff, the concentration of analytes is slightly higher than LoD and ≥95% samples should yield positive results (n ≥ 20). For moderate/strong positive samples, 100% samples should give positive results with CV ≤15% (n ≥ 20).

What should be noted that precision study described in EP15-A3 is not suitable for the precision evaluation of the SARS-CoV-2 tests. The protocol described in EP15-A3 is used to verify a manufacturer’s claims for precision and the trueness of the measurement procedure relative to the assigned values of materials with known concentrations [45], but not to establish or validate the precision performance of a measurement procedure, which has been described in EP05-A3 [44].

### Hook effect study

The hook effect is observed in sandwich immunoassays, where at very high concentrations of the analyte, the assay signal is saturated and leveled off [46].

It is necessary to detect gradient dilutions diluted from multiple high-concentration samples to evaluate the hook effect. Three to five duplicates for each dilution should be tested. The results of the hook effect should be clearly indicated on the instructions for use.

### Verification of reference materials

Three batches of products should be used to test the reference materials produced by the manufacturer described in the ‘Research data of main raw materials’ section. Considering the differences of materials, time, environment, operators and other occasional factors, the performance maybe different among batches. It is the common way to evaluate the performance of *in vitro* diagnostic (IVD) products using three or more batches to reduce the deviation caused by different batches, which is also described in the ‘The announcement of registration document requirements and the approved Certificate format for *in vitro* diagnostic reagents' (2014-09-05)’ [47]. Comprehensive experimental data of the verification should be provided.

### Instrument used for SARS-CoV-2 tests

In China, an IVD reagent and instrument used together as a system are registered separately as a different product and the matched instrument is required to be listed in the instructions for the use of the IVD reagent. It is the same for SARS-CoV-2 antigen/antibody tests that all of the instruments used for result reading and interpretation should be listed in the product instructions, and the analytical performance evaluation using each instrument should be provided.

### Specimen types

If serum and plasma are the applicable specimen types for the product, the comparability between them can be verified by homologous comparison method. For whole blood, two methods can be chosen for evaluation, one is to study all of the analytical performance and another is to analyze at least the LoD, inclusivity of virus samples from different regions, precision and the homologous comparison test between different specimen types. The sample collection is suggested to be conducted in accordance with the ‘*Technical Guidelines for Laboratory Detection of SARS-CoV-2-infected Pneumonia*’ [17].

## Determination of cut-off value

It is required to provide the research data that determines the cut-off value for positive and negative results or a zone where the response is ‘indeterminate’, which can be referred to EP28-A3c [48]. Different geographic regions, different infection stages and physiological status should be considered when selecting samples for the establishment of the cut-off. Moreover, differences among sample types should be clarified and confirmed respectively. The receiver operating characteristic curve is recommended to determine the cut-off, and the equivocal zone should be investigated if it exists.

## Research data of main raw materials, production process & reaction system

The main raw materials of this product include antigen, antibody, quality control materials and reference materials. It is required to provide the research data on the selection and source, preparation process and quality standard of main raw materials. For the main production process, the principle of reaction should be introduced and the main production process should be clarified. The flow chart can be used to make it clear.

The establishment, optimization and confirmation of reaction system are required. The contents include reaction time, reaction temperature, washing liquid volume and washing times (if involved), as well as loading method and loading amount, should be optimized. As to IgM antibody assay with indirect method, the interference of high-concentration-specific SARS-CoV-2 IgG on IgM results should be considered and the reasonable sample treatment should be designed to reduce this interference.

## Evaluation of stability

Research data on stability mainly involves two parts, the stability of the reagent and the stability of applicable samples. The stability of applied reagent mainly include shelf-life stability, in-use stability and accelerated stability under damage of high temperature and transport simulation [49]. For shelf-life stability research, at least three lots of products are required to be evaluated. The sample stability mainly focuses on the validity of specimens under refrigerating and freezing conditions. Freeze–thaw effects should be evaluated if applicable. The treated samples, such as samples in preservation solution, should be studied. Moreover, if heated samples (e.g., thermal inactivation) are used for detection, the interference of heating factor should be verified for positive samples with SARS-CoV-2 specific IgM antibody before and after being heated. No less than five positive heated and unheated samples near the cut-off value should be compared.

## Clinical studies

The clinical trial for the SARS-CoV-2 antigen/antibody tests should be conducted in at least three clinical sites (including Centers for Disease Control and Prevention) as required, which helps to ensure the diversity of the cases enrolled in the clinical trial [50]. Ideally, the clinical performance of SARS-CoV-2 antibody tests should be evaluated by comparing the tests with the results of the available clinical reference standard for COVID-19. Additionally, the clinical performance of SARS-CoV-2 antigen tests should be evaluated by comparing the tests with the results of the synchronous SARS-CoV-2 nucleic acid tests.

### Determination of clinical reference standard

The SARS-CoV-2 antigen/antibody test is generally expected to be used for the auxiliary diagnosis of COVID-19. Therefore, in clinical trials, the clinical diagnosis criteria for COVID-19 and the judgement criteria of disease progression shall be used as reference in the comparative study of SARS-CoV-2 antibody tests. ‘*Diagnosis and Treatment Protocol for Novel Coronavirus Pneumonia*’ and other documents issued by the National Health Commission of the People's Republic of China have specified the diagnosis criteria for COVID-19 [13]. It is recommended to compare the positive detection rate of antibody test to that of the nucleic acid test of COVID-19 in order to fully evaluate the clinical significance of antibody tests. For the clinical trial of the SARS-CoV-2 antigen tests, the SARS-CoV-2 nucleic acid tests will be the preferred comparator and the diagnosis criteria for COVID-19 will help to evaluate the clinical performance more scientifically and explain the inconsistent result.

### Study population & inclusion criteria

Subjects or samples enrolled in the clinical trial should represent the target population of the test. The studied population of SARS-CoV-2 antigen or antibody tests should be the COVID-19 suspected cases. The definition of ‘suspected case’ can refer to the ‘*Diagnosis and Treatment Protocol for Novel Coronavirus Pneumonia*’ [13] issued by the National Health Committee of the People’s Republic of China.

The subjects enrolled in the clinical trial should represent various types of target population, including the confirmed cases of COVID-19 (including some cases in recovery period) and excluded cases. For the antibody tests, the confirmed cases should include the patients in different course of disease, and for the antigen tests, the confirmed cases are suggested to be mainly of the patients in the early stage of infection.

Moreover, for the evaluation of SARS-CoV-2 antibody tests, it is required to include some subjects with multi-time-point surveillance data of different stage of disease, where SARS-CoV-2 nucleic acid and antibody transformation can be detected.

### Sample types for clinical trial

Generally, the sample types of SARS-CoV-2 antibody detection reagent are serum, plasma and whole blood. The sample types of SARS-CoV-2 antigen detection reagent are throat swab and other respiratory samples. The sample collection is suggested to be conducted in accordance with the ‘Technical Guidelines for Laboratory Detection of SARS-CoV-2-infected Pneumonia’ [17].

For different sample types, if it is proved that there is no difference in analytical performance (e.g., there is no difference between serum and plasma samples in analytical performance), the detection data for different types of sample can be analyzed and summarized together or homologous samples comparison can be conducted in clinical trials. In other cases, statistical analysis for each type of sample is recommended and the requirements for the number of cases should be met respectively.

### Sample size for clinical trial

At present, the developed SARS-CoV-2 antigen/antibody test is qualitative detection product generally. In the process of clinical trial, it is suggested to estimate the minimum sample size using a reasonable statistical model according to the preset clinical evaluation measures, such as clinical sensitivity, clinical specificity and relevant statistical parameters. Among them, the clinical evaluation measures should be confirmed in accordance with the previous studies of this product. For instance, if the expected clinical sensitivity is 85%, the confirmed cases are estimated to be no less than 200 cases according to the sample size calculation formula for sampling precision. If the expected specificity can reach 95% and according to the clinical requirements, the specificity of such tests should be no less than 90%, based on the sample size calculation formula for single arm study with performance goal, the excluded cases enrolled should be no less than 300. Applicant may estimate the sample size according to the specific characteristics of product.

In order to conduct more scientific and comprehensive evaluation of clinical performance of product, a certain number of patients at different stages of disease should be included for the SARS-CoV-2 antibody tests. When necessary, the sample size of each important subgroup should meet the statistical requirements.

### Statistical analysis of clinical trial results

The aim of the clinical trial for the SARS-CoV-2 antibody tests is to verify the consistency between the test product and the clinical reference standard. The test results are generally summarized in the form of table 2 × 2, by which the clinical sensitivity, clinical specificity and confidence interval are calculated.

For the SARS-CoV-2 antibody tests, stratification analysis should be conducted for the patients in different stages of disease course. For the multi-time-point surveillance samples of COVID-19 patients for antibody tests evaluation, it is required to compare the test results of nucleic acid simultaneously and evaluate the detection capability and window period of the antibody reagent for trial for SARS-CoV-2 infection.

In order to evaluate the clinical significance of IgG and IgM joint detection, in addition to separate statistics for IgG and IgM test results, a statistical analysis of comprehensive evaluation of the two indicators should also be performed.

All inconsistent results in the clinical trial should be analyzed fully by combining them with the epidemiological background, clinical symptoms, prognosis of disease and other information of patients.

### Formal requirements for clinical evidences

In accordance with the requirements of ‘Provision of In-Vitro Diagnostic Reagent Registration’ [15], ‘The announcement of registration document requirements and the approved Certificate format for *in vitro* diagnostic reagents’ [47] and other regulatory documents, applicant should submit ethical review opinions of all institutions, clinical trial protocol, clinical trial report and summary report on clinical trial.

The summary sheet of clinical trial data should be submitted as the attachment of clinical trial report. The data sheet should include the record number, age, gender, sample type, sampling time, background information on clinical diagnosis, detection results of this product and the confirm or exclusion results of SARS-CoV-2 infection. Meanwhile, it is required to specify the nucleic acid test results (including the name of nucleic acid test reagent) used for the diagnosis of the disease. All the data in clinical trials should be traceable. Considering that the generation of antigen and antibody is closely related to the course of pathogen infection, it is suggested to specify the time of disease onset, the changes of symptoms and the disease prognosis of patients in clinical background information.

## Conclusion

The Key Points for the SARS-CoV-2 test was issued to facilitate the availability of SARS-CoV-2 antigen and antibody tests to respond to the COVID-19 public health emergency effectively. It describes the requirements for the SARS-CoV-2 antigen and antibody tests for emergency use during the COVID-19 pandemic. It not only facilitates the reviewer to review such products under consistent standard and ensure safety, effectiveness and controllable quality of products in a short period, but also provides a guidance for the enterprise to develop the SARS-CoV-2 diagnostic products and prepare the application for approval. Although this Key Points was issued at the beginning of this pandemic, its principle is still applied for the situation. With further understanding of COVID-19 and SARS-CoV-2, new requirements may be needed for new situations and the Key Points may be revised accordingly.

## Future perspective

Since the global outbreak of COVID-19, more than one wave of COVID-19 infections and different mutations of SARS-CoV-2 have been reported in different regions [24] and the spread of SARS-CoV-2 has not been controlled yet. The upcoming 2020–2021 flu season will bring more challenge to the prevention and control of SARS-CoV-2 infection. The accurate, specific and rapid SARS-CoV-2 tests and the combined detection of the potential coinfection are critically important for making effective measurements to respond and control the SARS-CoV-2 and other infections. SARS-CoV-2 antibody tests could provide useful information on the infection of the population, especially when SARS-CoV-2 RNA cannot be detected, but positive results of antibody tests cannot be used to confirm the presence of the virus. SARS-CoV-2 antigen tests undergone stringent regulatory review is much less than nucleic acid and antibody tests. The rapid detection of SARS-CoV-2 antigen displays the potential merit of ease-usage, rapid turnaround time and wide range of application due to decentralized testing of patients, which may facilitate the diagnosis of patients with early symptoms and the guidance for antigen detection in the diagnosis of SARS-CoV-2 infection using rapid immunoassays has been provided by WHO [51]. Comprehensive understanding of the dynamic evolution of SARS-CoV-2 antibody and antigen will facilitate to the design of accurate, sensitive and specific SARS-CoV-2 tests, and help to choose a reasonable detection time and frequency. Normalized prevention and countermeasures, hierarchical management and immediate response will facilitate the early control of regional outbreak. Systematic strategy for prevention, control and treatment and effort from the whole world are needed to fight against this global COVID-19 pandemic.

**Executive summary**

**Background**

- Coronavirus disease-2019 (COVID-19) caused by severe acute respiratory syndrome coronavirus 2 (SARS-CoV-2) has spread globally and more and more waves of COVID-19 infections have occurred in different regions. The coming winter and flu infection will make the control and treatment of COVID-19 pandemic more complicated. SARS-CoV-2 antigen/antibody tests, combined with SARS-CoV-2 nucleic acid tests with high efficiency and rapid accessibility will play an important role in prevention and treatment for this continuous pandemic. The purpose of this article is to provide more explanation on the requirement for the SARS-CoV-2 antigen/antibody tests to facilitate the development and accessibility of such tests.

**Scope of application**

- The Key Points are applicable for SARS-CoV-2 antigen/antibody assays, which are used to conduct *in vitro* qualitative detection of SARS-CoV-2 antigen/antibody in the samples of serum, plasma, whole blood, throat swab, bronchoalveolar lavage fluid, sputum or other respiratory secretions.

**Analytical performance evaluation**

- All analytical performance of candidate tests should be evaluated and verified using reagent manufactured under the control of quality management system.

**Sample collection & processing**

- Disease course, clinical symptoms and medication treatment should be considered.

**Limit of detection**

- Evaluation of limit of detection and verification of limit of detection claim should be carried out with enough replicates and different samples using suitable analysis method.

**Inclusivity**

- At least ten different real clinical samples of COVID-19 patients from different sources with temporal and regional characteristics should be verified.

**Analytical specificity**

- Cross reactivity should be analyzed for other human coronaviruses, influenza virus and other common respiratory viruses or coinfections of other viruses with SARS-CoV-2. The interference of suitable endogenous/exogenous substances should be evaluated.

**IgM antibody class specificity**

- IgM damage study should be carried out to verify the specification of IgM test.

**Precision**

- Multiple factors and reasonable evaluation period should be considered for precision evaluation. Negative samples, positive samples near the cutoff and moderate/strong positive clinical samples should be analyzed.

**Hook effect study**

- The concentration of targets without hook effect should be clarified.

**Verification of reference materials**

- Three batches of products should be used to test the reference materials.

**Instrument used for SARS-CoV-2 tests**

- Instruments used by proposed tests should be evaluated and clarified in production instruction for use.

**Specimen types**

- Applicable specimen types should be verified using suitable method.

**Determination of cut-off value**

- Different geographic regions, different infection stages, physiological status and sample types should be considered for cut-off value determination. Receiver operating characteristic curves are recommended.

**Research data on main raw materials, production process & reaction system**

- It is required to provide the research data on the selection, source, preparation and quality standard of main raw materials, such as antigen, antibody, quality control materials and reference materials.

**Evaluation of stability**

- Reagent stability and sample stability should be evaluated. Three lots of reagents are needed for shelf-life stability. The stability of treated samples by different method (preservation solution or heating) should also be studied.

**Clinical studies**

- Antibody tests should be evaluated by comparing the results of the available clinical reference standard for COVID-19. And the clinical performance of SARS-CoV-2 antigen tests should be compared with the results of SARS-CoV-2 nucleic acid tests.

**Determination of clinical reference standard**

- The positive detection rate of tested kits to that of the COVID-19 nucleic acid test can be used to fully evaluate the clinical performance of antibody tests.

**Study population & inclusion criteria**

- Subjects or samples enrolled in the clinical trial should represent the proposed target population of the test.

**Sample types for clinical trial**

- Statistical analysis for each type of sample is recommended and the requirements for the number of cases should be met respectively. For serum and plasma, homologous sample comparison can be conducted.

**Sample size for clinical trial**

- It is suggested to estimate the minimum sample size using a reasonable statistical model according to the preset clinical evaluation measures, such as clinical sensitivity, clinical specificity and relevant statistical parameters.

**Statistical analysis of clinical trial results**

- For the SARS-CoV-2 antibody tests, stratification analysis should be conducted for the patients in different stages of disease course. The clinical significance of separate statistics for IgG and IgM and joint detection of IgG and IgM should be evaluated.

**Formal requirements for clinical evidences**

- Ethical review opinions of all institutions, clinical trial protocol, clinical trial report and summary report on clinical trial.

**Conclusion**

- The Key Points for the SARS-CoV-2 test was issued to facilitate the availability of SARS-CoV-2 antigen and antibodies tests to response the COVID-19 public health emergency effectively.
